# Immunogenicity of Comirnaty Omicron XBB.1.5 booster COVID-19 mRNA vaccine in long-term survivors after allogeneic hematopoietic stem cell transplantation

**DOI:** 10.1038/s41598-024-74712-x

**Published:** 2024-10-21

**Authors:** Sigrun Einarsdottir, Samer Al-Dury, Ellen Fridriksson, Linn Dahlsten Andius, Hao Wang, Sinan Sharba, Amin Mountagui, Johan Waern, Johan Ringlander, Anna Martner, Kristoffer Hellstrand, Jesper Waldenström, Martin Lagging

**Affiliations:** 1 Department of Hematology, Institute of Medicine, Sahlgrenska Academy, University of Gothenburg, Gothenburg, Sweden; 2 Department of Gastroenterology and Hepatology, Institute of Medicine, Sahlgrenska Academy, University of Gothenburg, Gothenburg, Sweden; 3 Department of Infectious Diseases, Institute of Biomedicine, Sahlgrenska Academy, University of Gothenburg, Gothenburg, Sweden; 4 Region Västra Götaland, Department of Clinical Microbiology, Sahlgrenska University Hospital, Gothenburg, Sweden; 5 TIMM Laboratory, Sahlgrenska Center for Cancer Research, Department of Infectious Diseases, Institute of Biomedicine, Sahlgrenska Academy, University of Gothenburg, Gothenburg, Sweden

**Keywords:** Allotransplantation, RNA vaccines

## Abstract

Primary mRNA vaccination against COVID-19 typically involves three doses for immunocompromised individuals, including hematopoietic stem cell transplantation (allo-HSCT) recipients. However, optimal subsequent boosting strategies remain unclear. This study aimed to assess the immunogenicity of a booster dose using the most recently updated vaccine (Comirnaty Omicron XBB.1.5) among long-term allo-HSCT survivors having previously received multiple mRNA vaccine doses, in median 4 (2–6). Thirty-four allo-HSCT recipients were enrolled at Sahlgrenska University Hospital, and peripheral blood samples were collected immediately before and four weeks after booster. Antibodies against the receptor-binding domain (anti-RBD) of spike 1 (S1) and nucleocapsid, as well as S1-specific ex vivo T-cell responses, were evaluated. Adverse events were monitored. Despite a median of 13 months since the prior vaccine dose, both humoral and T-cell responses against S1 were present in the pre-booster samples in all but two participants, who suffered from severe chronic Graft-versus-host disease. Notably, 62% of participants had a previously confirmed COVID-19 infection. Significantly higher pre-booster antibody levels were observed in women than men (*p* = 0.003). Booster dosing strengthened specific antibody and T cell responses and equalized pre-booster gender differences, although responses remained significantly lower among those receiving immunosuppressive treatment (*p* = 0.041). In a population of long-term allo-HSCT survivors, the majority of whom had a prior confirmed COVID-19 infection, both pre- and post-booster immune responses were robust. However, patients undergoing immunosuppressive treatment for GvHD exhibited significantly weaker responses.

## Introduction

Multiple studies have examined both immunogenicity and clinical efficacy of mRNA COVID-19 vaccines among recipients of allogeneic hematopoietic stem cell transplantation (allo-HSCT), confirming their pivotal role in preventing COVID-19 associated morbidity and mortality among fully vaccinated individuals^1,2^ despite lack of detectable seroconversion in 5–26% of recipients after three vaccine doses^3–5^. The emergence of the less virulent Omicron variants and the development of effective antiviral treatments have further ameliorated the course of COVID-19. Breakthrough infections, i.e., infection despite full immunization, are often mild^6^, but deaths have been reported among allo-HSCT recipients with poor humoral vaccine responses^7^. The primary vaccine schedule, according to guidelines from the European Conference for Infections in Leukemia (ECIL) and the Centers for Disease Control and Prevention (CDC) includes three doses, starting at 3–6 months post-transplantation^8–10^, but subsequent boosting strategies remain to be elucidated.

This study thus aimed at assessing the immunogenicity of a COVID-19 booster dose using the most recently updated vaccine (Comirnaty Omicron XBB.1.5) among long-term allo-HSCT survivors having previously received multiple mRNA vaccine doses, in median 4 doses (2–6).

## Methods

### Study population and design

This study was a sub-study within the DurIRVac study, conducted at Sahlgrenska University Hospital in Gothenburg, Sweden between March 2021 and November 2023. Allo-HSCT recipients were identified using local transplant registries. The cohort mainly consisted of long-time allo-HSCT survivors (baseline characteristics in Table 1), with a median time elapsed since transplant of 9 (range 1–18) years. The majority of patients (*n* = 30) were enrolled early in the pandemic, before the first vaccine dose, and four more patients were newly recruited. Thirteen patients (38%) had chronic Graft-versus-host disease (cGvHD) at the visit prior to vaccination, and 6 (18%) were receiving immunosuppressive therapy at the time of vaccination. A majority, 62%, had earlier COVID-19 infection, details in Table 1. Enrolled transplant recipients fulfilled the following predetermined criteria at inclusion (i) to be at least three months post allo-HSCT (ii) not to have received rituximab the last six months and, (iii) to be without uncontrolled acute GvHD (grade III to IV). All according to EBMT-criteria at study start (version 2.0, December 21, 2020).

**Table 1:** Patient characteristics.

Baseline characteristics.	All patients (*n* = 34)	No active cGvHD (*n* = 24)	Active cGvHD (*n* = 10)
**Diagnosis**			
Acute myeloid leukemia (AML)	14 (41%)	11 (46%)	3 (30%)
Acute lymphoblastic leukemia (ALL)	5 (15%)	3 (13%)	2 (20%)
Chronic myeloid leukemia (CML)	6 (18%)	3 (13%)	3 (30%)
Lymphoma	3 (9%)	1 (4%)	2 (20%)
Myelofibrosis	2 (6%)	2 (8%)	0
Other^2^	4 (12%)	4 (17%)	0
**Sex**			
Male	16 (47%)	11 (46%)	5 (50%)
Female	18 (53%)	13 (54%)	5 (50%)
**Reduced intensity (RIC) or Myeloablative conditioning (MAC)**			
RIC	15 (44%)	11 (46%)	4 (40%)
MAC	19 (56%)	13 (54%)	6 (60%)
**cGvHD-severity**			
Mild	4 (12%)		4 (40%)
Moderate	3 (9%)		3 (30%)
Severe	3 (9%)		3 (30%)
**Ongoing GvHD-therapy at vaccination**			
Systemic IST^3^	6 (18%)		6 (60%)
Topical	3 (9%)		3 (30%)
No ongoing GvHD-therapy	25 (71%)	24 (100%)	1 (10%)
**Previous COVID-19 infection**			
Confirmed by PCR	7 (21%)	5 (21%)	2 (20%)
Symptoms + positive antigen test	8 (24%)	4 (17%)	4 (40%)
Positive nIgG only	6 (18%)	3 (13%)	3 (30%)
A combination of above	6 (18%)	3 (13%)	3 (30%)
**Vaccination details**			
Age at vaccination in years (median, min-max)	56 (30–81)	62 (30–81)	44 (32–79)
Time since last vaccine dose (months: median, min-max)	13 (2–30)	12 (2–23)	13 (3–30)
Number of previous vaccine doses (median, min-max)	4 (2^1^-6)	4 (2–5)	4 (2^1^-6)
Previous COVID-19 mRNA vaccine (BNT162b2 (Pfizer-BioBTech Comirnaty)/ mRNA-1273 (Moderna Spikevax)/mix of both vaccines/not sure	18/3/5/8	10/2/5/7	8/1/0/1
Time from transplant at booster (months: median, min-max)	101 (11–277)	101 (11–277)	101 (34–249)

^1^One patient had received two doses post transplantation but four doses pre transplantation.

^2^Aplastic anemia (1), CVID (1), Chronic myelomonocytic leukemia (CMML) (1) and Myelodysplastic syndrome (MDS) (1).

^3^prednisone (*n* = 6), ruxolitinib (*n* = 6), and photopheresis (*n* = 3). All patients were on combinations of immunosuppressants.

Patients were invited to receive the booster at Sahlgrenska University Hospital in November 2023. Peripheral blood was collected immediately before and 4 weeks after the booster immunization. Currently, all participants received the most recently updated Pfizer-BioNTech mRNA vaccine, Comirnaty Omicron XBB.1.5. All allo-HSCT recipients completed a standardized questionnaire following vaccination regarding side effects, which were categorized per the Common Terminology Criteria for Adverse Events standards. Information about GvHD using the National Institutes of Health global severity score was assessed through the medical notes from the visit prior to vaccination.

### Analysis of IgG against the RDB within S1 of SARS-CoV-2 (anti-RBD-S1 IgG) and nucleocapsid of SARS-Cov-2

Chemiluminescent microparticle immunoassays (CMIAs) were performed using the automated Alinity system for analysis of IgG antibodies against the receptor-binding domain (RBD) (SARS-CoV-2 IgG II Quant, Abbott, Illinois, USA) within the spike protein (anti-RBD IgG). Levels were reported in the WHO international standard binding antibody units (BAU)/mL (detection range of 14-5680 BAU/mL). Samples above 5680 BAU/mL were diluted and reanalyzed. Antibodies against nucleocapsid (SARS-CoV-2 IgG, Abbott, Abbott Park, Illinois, USA) were also evaluated (nIgG).

## Assay of peptide-induced cytokine release in whole blood

Whole blood was stimulated with 1 µg/mL/peptide of 15-mer peptides with 11-amino acid overlap spanning the N-terminal S1 domain of the SARS-CoV-2 surface glycoprotein (S1; product number: 130-127-041, Miltenyi Biotec). After incubation at 37 °C plasma and 5% CO_2_ for two days, plasma was recovered for analysis of IFN-γ and IL-2 using ELISA (3420–1 H-20 and 3445-1 H-20; Mabtech AB). The S1-induced IFN-γ and IL-2 production is presented with levels in PBS-stimulated samples subtracted. The limit of detection was 10 pg/mL. All methods were performed in accordance with the relevant guidelines and regulations.

## Documentation of COVID-19

Previous COVID-19 infection was defined as one or several of the following: detectable SARS-CoV-2 RNA, reactive COVID-19 antigen test, and/or presence of antibodies against the nucleocapsid. Twenty-one participants (62%) had a documented COVID-19 infection at some point during the study period before pre-booster sample.

### Statistical analysis

Continuous variables were described as median and range, that is, min-max values, as applicable. Categorical data were described with contingency tables including frequency and percent. The association between continuous parameters was determined using Spearman’s correlation. Statistical comparisons between pre- and post-booster samples were calculated by Wilcoxon matched-pairs signed-rank test. The association between various parameters in independent groups was performed by Mann-Whitney U-test. Statistical analyses were performed using SPSS statistical software package (version 28) or GraphPad Prism software (version 9). P-values are designated as follows: *P < 0.05, **P < 0.01, and ***P < 0.001. All indicated P-values are 2-sided.

## Ethical considerations and trial registration

All participants gave written informed consent prior to enrollment. The DurIRVac study was approved by the Swedish Ethical Review Authority (permit nos. 2020–03276, 2021 − 00374, and 2021 − 00539) and by the Swedish Medical Products Agency (Dnr: 5.1-2021-11118) and has been registered at the European Union Drug Regulating Authorities Clinical Trials Database (EudraCT no. 2021-000349-42).

## Results

### Humoral booster immune responses

**Fig. 1:**
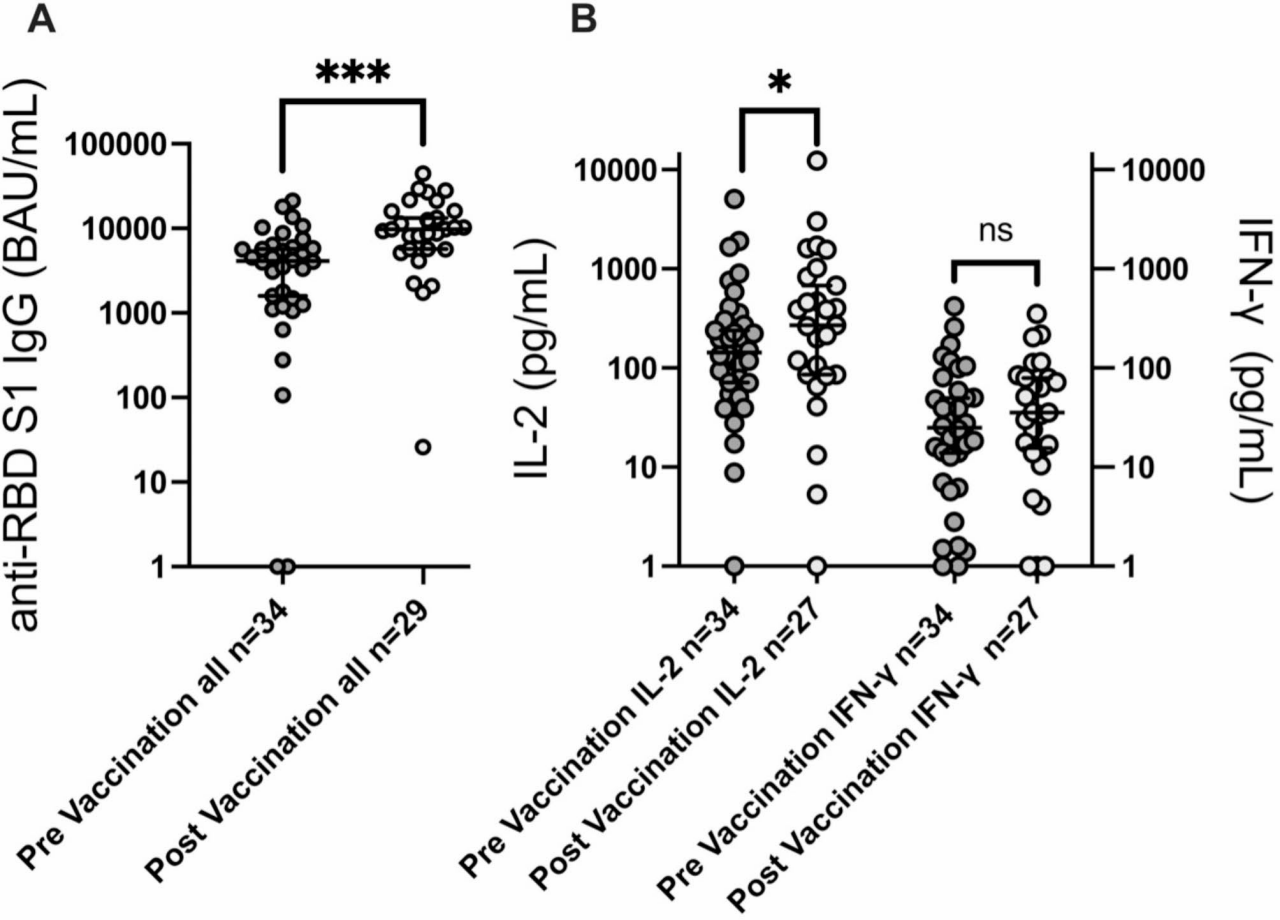
Booster vaccination induces enhanced serological and specific T-cell responses to spike 1 of SARS-CoV-2 in allo-HSCT recipients. (A) shows serum levels of immunoglobulin G (IgG) against the receptor binding domain (RBD) of spike 1. (B) shows IL-2 and interferon-γ (IFN-γ) production in supernatant plasma following stimulation of whole blood with spike 1 peptides, reflecting the reactivity of SARS-Cov-2 specific T-cells. Median with 95% confidence interval. Statistical comparisons by Wilcoxon matched-pairs signed-rank test. P-values are two-sided.

**Fig. 2:**
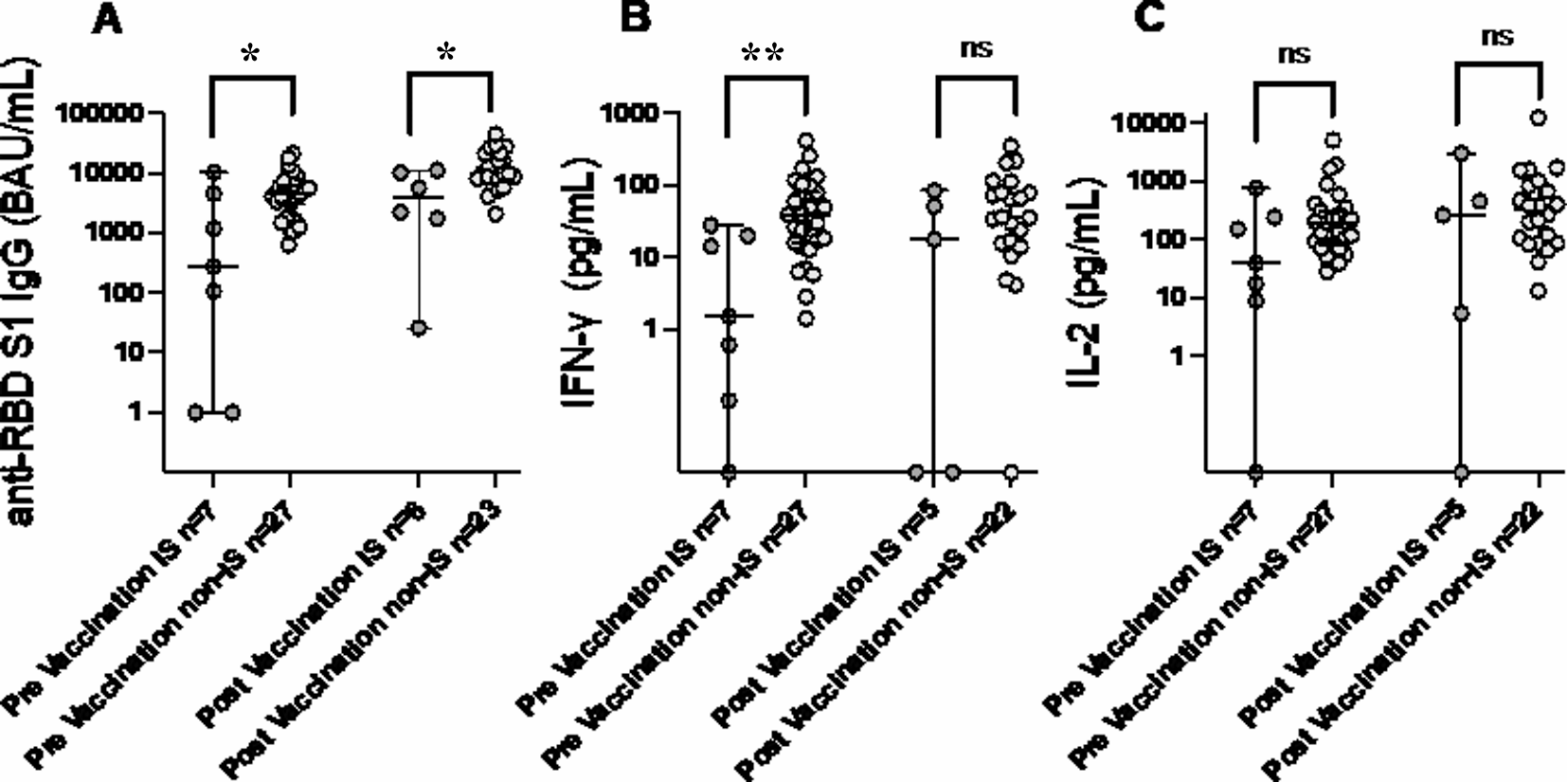
Reduced spike 1 specific immune responses in allo-HSCT recipients on immunosuppressive treatment. (A) shows serum levels of immunoglobulin G (IgG) against the receptor binding domain (RBD) of spike 1 in patients on immunosuppression (IS) compared to patients not receiving immunosuppression (non-IS). (B) shows interferon-γ (IFN-γ) and (C) shows IL-2 production in plasma supernatant following stimulation of whole blood with spike 1 peptides patients on immunosuppression compared to patients not receiving immunosuppression. Median with 95% confidence interval. Statistical comparisons by Mann-Whitney U-test. P-values are two-sided.

**Fig. 3:**
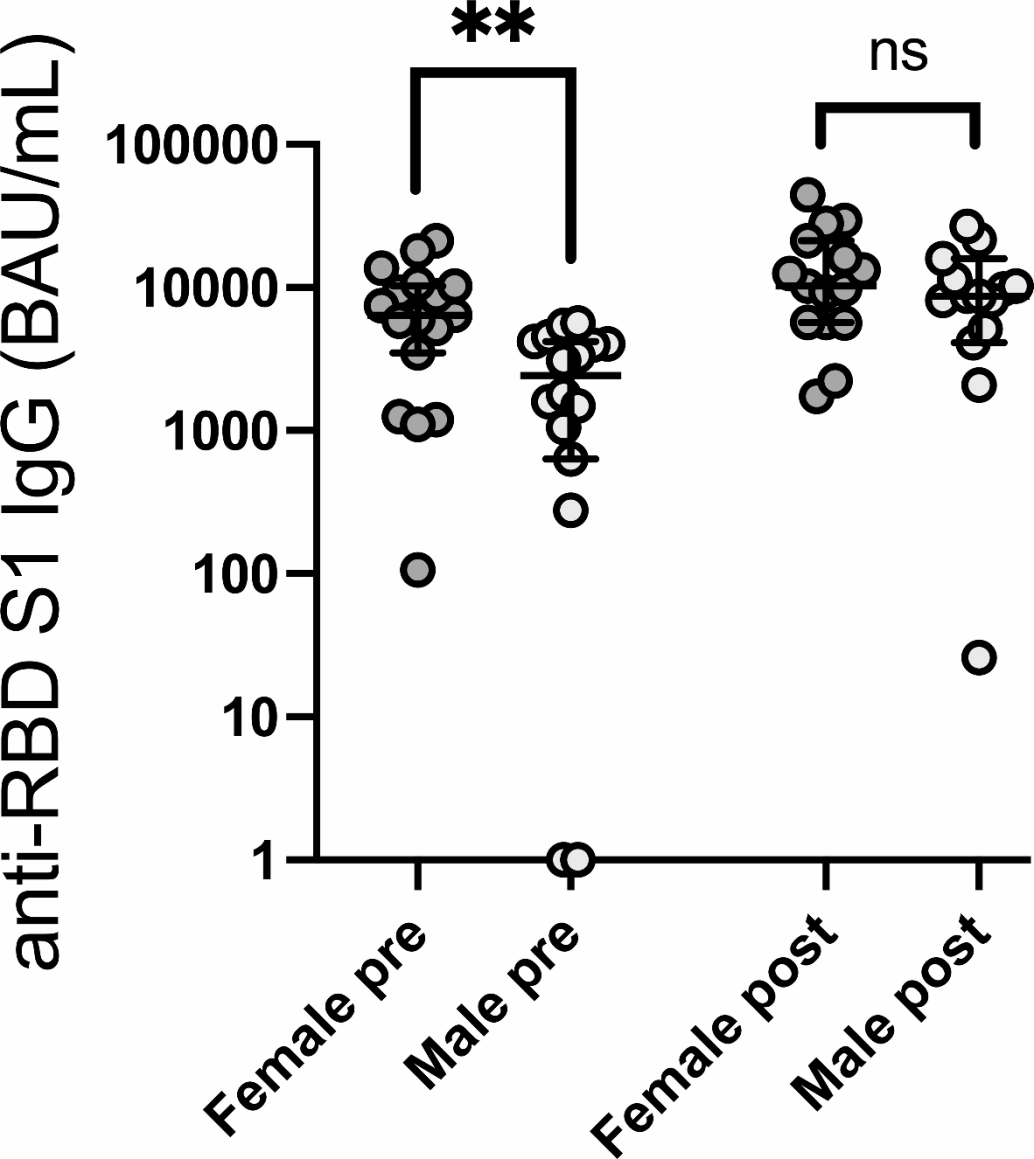
Reduced serological response to spike 1 in male allo-HSCT recipients prior to booster vaccination. Serological response (immunoglobulin G (IgG) against the receptor binding domain (RBD) in spike 1 before and after a booster mRNA COVID-19 vaccine dose delivered to allo-HSCT recipients comparing responses in males and females. Median with 95% confidence interval. Statistical comparisons by Mann-Whitney U-test. P-values are two-sided.

Following mRNA vaccine boosting, anti-RBD IgG levels increased from a median of 4123 (0-21291) to 9718 (26-44436) BAU/mL (*p* < 0.0001; Fig. 1). Patients with ongoing immunosuppression had significantly lower pre-booster (median 278 vs. 4513 BAU/mL; *p* = 0.019) as well as post-booster anti-RBD concentrations (median 4513 vs. 9780 BAU/mL; *p* = 0.041; Fig. 2). Two patients lacked detectable antibody levels pre-booster, both of whom were receiving immunosuppressive treatment for severe chronic GvHD, but all had quantifiable concentrations post-booster. Clinical details on the seronegative patients are provided in Table 2. Female allo-HSCT recipients had significantly higher pre-booster (median 6403 vs. 2430 BAU/mL for female vs. male participants respectively; *p* = 0.003) but not post-booster anti-RBD IgG antibody levels (*p* = 0.3; Fig. 3) in comparison to male allo-HSCT recipients. In contrast, the number of previous vaccine doses, previous COVID-19 infection, time elapsed since transplantation, time since last vaccine dose, and age were not significantly associated with pre- or post-booster humoral responses, although this may be secondary to the relatively small sample size.

**Table 2:** Clinical details on the two seronegative patients prior to booster.

	Patient 1	Patient 2
Age at vaccination	49	48
Sex	M	M
Time since last vaccine dose (months)	1.5	19
Time since transplant at booster (months)	11	38
Number of previous vaccine doses post allo-HSCT	2	4
Previous COVID-19 infection	no	yes
Number of vaccine doses prior to allo-HSCT	4	0
Diagnosis	AML^1^	MCL^2^
Conditioning intensity	MAC^3^	MAC
Stem cell donor	MMRD^4^ (haplo)	URD (10/10)^5^
Stem cell source	BM^6^	PB^7^
cGvHD^8^	Severe extensive	Severe extensive
Immunosuppression	Ruxolitinib 10mgx2, Prednisone 5mgx1	Ruxolitinib 10mgx2 Prednisone 5mgx1 Photopheresis 2/2weeks
sIgG prior to booster	0	0
sIgG post booster	26	10 286
nIgG prior to booster	0	0
nIgG post booster	0	0
T-cell reactivity prior to booster (IL-2)	8.3	39.3
T-cell reactivity post booster (IL-2)	5.3	3004.8
T-cell reactivity prior to booster (IFN-y)	0	1.5
T-cell reactivity post booster (IFN-y)	0	31.4
Adverse events following vaccination	none	none

^1^ Acute myeloid leukemia, ^2^Mantle cell lymphoma, ^3^Myeloablative conditioning, ^4^Mismatched related donor, ^5^Unrelated donor, ^6^Peripheral blood, ^7^Bone marrow, ^8^cGvHD: chronic Graft-versus-host disease.

## T cell immune booster responses

S1-specific IL-2 levels significantly increased from a median of 143 (0-5079) to 269 (0-12304) pg/mL (*p* = 0.038), while IFN-γ release was less affected by the booster (30 (0-418) to 35 (0-352); *p* = 0.83; Fig. 1). Neither the number of previous vaccine doses, previous COVID-19 infection, time since transplantation, time since last vaccine dose or age were significantly associated with pre-booster or post-booster ex vivo T-cell responses.

### Correlation between humoral and T cell immune booster responses

Pre-booster anti-RBD IgG level was correlated with pre-booster T-cell reactivity, both with regards to S1-specific IL-2 levels (rho = 0.39, *p* = 0.023) and IFN-γ production (rho = 0.53, *p* = 0.001). Post-booster anti-RBD IgG antibody concentrations were correlated with post-booster IFN-γ production (rho = 0.39, *p* = 0.045) but not with S1-specific IL-2 levels (rho = 0.26, *p* = 0.19).

Pre-booster anti-RBD IgG was also correlated with post-booster anti-RBD IgG (*p* = 0.0015), post-booster IL-2 (*p* = 0.003) and post-booster IFN-γ (rho = 0.5, *p* = 0.09). Post-booster anti-RBD IgG was also correlated with pre-booster levels of IFN-γ (rho = 0.49, *p* = 0.007).

### Impact of recent COVID-19 infection and immune responses

Antibodies against nucleocapsid (nIgG) were noted in 12/34 (35%) allo-HSCT recipients, indicating probable exposure to SARS-CoV-2 within the past year^11^, but did not impact on pre- or post-booster anti-RBD IgG levels, ex vivo T-cell responses, need for hospitalization nor documented respiratory illness the past 6 months.

### Adverse events

Fourteen of 25 (56%) patients reported side effects, of whom 7/14 (50%) reported only local side effects and 7/12 (50%) reported systemic side effects, mainly fever and malaise. No severe adverse events or worsening of GvHD were reported.

## Discussion

The main finding in our study was that among these long-term allo-HSCT survivors, both humoral B-cell and ex vivo T-cell responses were robust in the pre-booster samples, despite almost a year having elapsed since the last vaccine dose. This robustness is likely attributable to the fact that most patients had previously contracted COVID-19, with 62% having a confirmed prior SARS-CoV-2 infection. It is probable that an even larger proportion had subclinical infections, with nucleocapsid antibodies waning below the detection threshold prior to sampling. Additionally, booster dosing with Comirnaty Omicron XBB.1.5 further strengthened virus specific immune responses, though responses were significantly lower both pre- and post-boosting among those receiving immunosuppressive therapy.

There was no correlation between earlier confirmed SARS-CoV-2 infection and antibody levels or T-cell responses, which might be explained by the very high frequency of previous COVID-19 and an acquired infection-vaccine hybrid immunity, which has been documented to enhance immune responses also among immunocompromised individuals^12,13^. During the first waves of the pandemic, SARS-CoV-2 PCR was available to the public through self-testing and both in- and outpatient were extensively tested, however, after late 2022 SARS-CoV-2 testing has mainly been limited to individuals at risk for severe disease and early intervention or already hospitalized. 35% of participants in this study had antibodies against the nucleocapsid (nIgG), which is in line with reports from the Swedish Public Health Agency suggesting periods of high transmission during 2023^14^, although with substantially attenuated clinical implications as compared to the early phases of the pandemic among allo-HSCT recipients^15^.

An increase in IL-2 following booster vaccination was noted, however, there was no significant change in IFN-γ production following booster. This finding is congruent with other studies investigating the cytokine profile following COVID-19 mRNA vaccination, where IL-2 is the dominant cytokine elicited by SARS-CoV-2 specific CD4 + cells post-immunization^16^.

A significant gender difference was also noted in pre-booster humoral immune responses among long-term allo-HSCT survivors, with female participants exhibiting higher antibody levels compared with their male counterparts, despite no differences in the number of previous doses, the time elapsed since the last dose or presence of cGvHD. This observation aligns with a growing body of research suggesting that gender can influence immune response to vaccinations^17^.

The Swedish Public Health Agency recommends booster COVID-19 mRNA vaccine doses for immunocompromised patients once to twice annually but acknowledge that this population is heterogeneous, and that the responsible physician is to advise on the boosting strategy. Some centers have opted to check serum antibody levels after the primary vaccination schedule to guide the need for subsequent reiterative doses. Tailored vaccination schemes, though promising, pose challenges due to the absence of clearly delineated thresholds regarding correlates of protection against severe COVID-19. Furthermore, the implementation of immune-guided vaccination strategies necessitates consensus on sampling schedules. Additionally, considerations regarding the inclusion of comorbidities, planned immunosuppressive treatments or the emergence of novel SARS-CoV-2 variants complicate decision-making processes in this regard. To this end, the finding in this study of high pre-booster immune responses, despite considerable time since the previous vaccine dose, in most patients is encouraging. The exception is long-term allo-HSCT survivors with ongoing immunosuppressive treatment, among who the COVID-19 specific immune responses were reduced.

Strengths with the current study include the parallel monitoring of aspects of virus-specific B- and T-cell–mediated immunity and sampling of participants right before the booster and at four weeks after. The study sample size was relatively small and, of presumable importance for outcomes, the median time from transplantation was nine years. With these precautions, we believe that our findings are applicable to long-term allo-HSCT survivors. There was no apparent impact of GvHD, but patients on immunosuppressive treatment, in our study mainly low doses of prednisone combined with ruxolitinib, had significantly lower immune responses.

One patient was on Ig replacement, this patient also had previous COVID-19 confirmed with both PCR and nIgG. He had high antibody levels, both in the pre booster and post booster sampling, but had a fivefold increase in sIgG after vs. before booster and a three-fold increase in IL-2, suggesting that his Ig replacement did not substantially confound results.

In conclusion, boosting strategies in immunocompromised patients remains challenging given the heterogenicity of this population. Among these long-term allo-HSCT survivors, most of whom had previously contracted COVID-19, the majority exhibited strong immune responses both pre- and post-booster. However, an exception was observed in allo-HSCT recipients undergoing immunosuppressive treatment, who had reduced COVID-19-specific immune responses before and after the booster.

## Data Availability

For original data, please contact the corresponding author. As per Swedish law, individual participant data will not be shared.
